# Efficient identification of mutations that potentially confer treatment resistance through computational derivation

**DOI:** 10.1186/1471-2105-16-S15-P5

**Published:** 2015-10-23

**Authors:** Christy M Gearheart, James R Lambert, Christopher C Porter

**Affiliations:** 1 Department of Pediatrics, University of Colorado School of Medicine, Aurora, CO 80045, USA; 2 Department of Pathology, University of Colorado School of Medicine, Aurora, CO 80045, USA

## Background

One of the major factors in cancer progression or relapse in patients is the development of chemoresistance. The heterogeneity of cancer subtypes often means that the oncogene addictions are different between subtypes, possibly between patients. We have developed an efficient computational pipeline to filter, analyze, and interpret the vast number of called mutations that differentiate between genomes to identify a targeted list of genetic determinants of phenotype; in this case drug resistant vs. sensitive cancer cells.

## Materials and methods

Called mutations are filtered for quality, depth coverage, and frequency of the mutation in the mapped reads. Only exonic regions are considered for further analysis. To quickly eliminate mutations in a resistant sample that are also present within the paired sensitive sample, we created an efficient algorithm that dramatically reduces the required search space. This is accomplished through the creation of a hash table mapping to a balanced binary tree of the individual chromosome mutation positions and base changes of the sensitive parental line, reducing the search space required to less than 1% of a brute force algorithm approach. Subsequent analysis of the remaining mutations interprets potential impact through two methodologies. The first method predicts if the specific mutation will have a synonymous or deleterious effect on the gene expression; the second method examines the mutations in aggregate for an individual gene under the hypothesis that highly mutated genes have a higher probability of disrupting functionality.

## Results

We have applied our bioinformatics pipeline to triple negative breast cancer (TNBC) cells to identify mutations that confer resistance to AMPI-109. AMPI-109 is an internally developed drug molecule that potently induces apoptosis in TNBC cells of various molecular subtypes, yet has little to no effect on non-TNBC and non-tumorigenic cell lines. To identify potential mechanisms of resistance, we compared the transcriptome of the sensitive TNBC BT-20 parental cell line against five AMPI-109 resistant clones. Validation of the targeted mutations identified from our pipeline are ongoing.

## Conclusions

This work establishes an efficient computational pipeline to quickly identify a targeted list of mutations potentially enabling chemoresistance in cancer patients. Early detection of mechanisms of resistance may enable more personalized therapeutic treatments with improved efficacy and survival. Moreover, this pipeline may be applied to any massively parallel sequencing application, such as the identification of somatic mutations in diseased tissue.

